# Universal Health Coverage for Non-communicable Diseases and Health Equity: Lessons From Australian Primary Healthcare

**DOI:** 10.34172/ijhpm.2020.232

**Published:** 2020-11-23

**Authors:** Matthew Fisher, Toby Freeman, Tamara Mackean, Sharon Friel, Fran Baum

**Affiliations:** ^1^Southgate Institute for Health, Society and Equity, Flinders University, Adelaide, SA, Australia.; ^2^College of Medicine and Public Health, Flinders University, Adelaide, SA, Australia.; ^3^The George Institute for Global Health, Sydney, NSW, Australia.; ^4^RegNet School of Regulation and Global Governance, Australian National University, Canberra, ACT, Australia.

**Keywords:** Australia, Universal Health Coverage, Primary Healthcare, Health Equity, Non-communicable Disease

## Abstract

**Background:** Universal health coverage (UHC) is central to current international debate on health policy. The primary healthcare (PHC) system is crucial to achieving UHC, in order to address the rising incidence of non-communicable diseases (NCDs) more effectively and equitably. In this paper, we examine the Australian case as a mature system of UHC and identify lessons for UHC policy to support equity of access to PHC and reduce NCDs.

**Methods:** Our qualitative research used policy mapping and monitoring and 30 key informant interviews, and applied policy theory, to investigate the implementation of Australian PHC policy between 2008 and 2018.

**Results:** Although the Australian PHC system does support equity of access to primary medical care, other ideational, actor-centred and structural features of policy detract from the capacities of the system to prevent and manage NCDs effectively, deliver equity of access according to need, and support equity in health outcomes. These features include a dominant focus on episodic primary medical care, which is a poor model of care for NCDs, and an inequitable distribution of these services. Also, a mixed system of public and private insurance coverage in PHC contributes to inequities in access and health outcomes, driving additional NCD demand into the health system.

**Conclusion:** Countries aiming to achieve UHC to support health equity and reduce NCDs can learn from strengths and weaknesses in the Australian system. We recommend a range of ideational, actor-centred and structural features of UHC systems in PHC that will support effective action on NCDs, equity of access to care according to need, and equity in health outcomes across geographically and ethnically diverse populations.

## Background

**Key Messages**

**Implications for policy makers**

- Australia holds lessons for global debate on universal health coverage (UHC) policies and the crucial role of primary healthcare (PHC), to address non-communicable diseases (NCDs) and promote health equity.
- Australia has a UHC system in PHC but it only partly supports equity and funds mainly episodic primary medical care; a poor model for NCDs.
- Australia’s mix of public and private insurance for UHC is also unfavourable to equity. Equitable UHC is most likely to be achieved in publicly funded and managed health systems.

**Implications for the public**

Primary healthcare (PHC) is the first level of healthcare people access for assistance with health needs. Universal PHC systems ensure that all people can get access to the care they need, regardless of income. Access to PHC can assist people to reduce their risk of non-communicable diseases (NCDs) such as heart disease, diabetes or mental illness; or manage those conditions when they occur. Currently, many governments are aiming to provide universal health coverage (UHC); that is, a health financing scheme to enable people to access healthcare – especially PHC – without suffering financial hardship. The paper reports on strengths and weaknesses of Australia’s UHC scheme, Medicare, in providing equity of access to PHC and reducing risk of NCDs; and identifies key lessons for other jurisdictions aiming to implement UHC schemes.

Universal health coverage (UHC) is central to current international health policy debate.^1-3^ The World Health Organization (WHO) defines UHC as when ‘all individuals and communities receive the health services they need without suffering financial hardship’ including ‘health promotion … prevention, treatment, rehabilitation, and palliative care.’^4^

 For countries without universal coverage, UHC schemes are likely to improve equity of access to health services.^5,6^ Primary healthcare (PHC) systems are fundamental to UHC.^1,7^ The WHO Director-General has added that, in contributing to UHC, PHC must address rising impacts of non-communicable diseases (NCDs), work to address social determinants of health (SDH), and place ‘communities at the centre of healthcare.’^1^ However, the focus of proposals for UHC is not on delivery of healthcare services as such but on financial instruments such as insurance schemes, which pool funds in order to partially or wholly cover individuals’ and families’ costs for using services.^2,8^ Furthermore, UHC schemes may include a role for private sector insurance services, or governments may choose to limit public coverage to selected services.^8^ Thus, UHC in practice may narrow governments’ aims to universal enrolment in an insurance scheme, limit services covered to selective primary care, or establish inequities between private and public schemes. Such outcomes would undermine long-standing WHO aims for universal access to comprehensive PHC.^2,5,8^

 Acknowledging international debate on UHC, Reich et al argue it is useful to consider the experience of countries that ‘have mature health systems with UHC’^9^; to identify pertinent insights for the debate or countries implementing UHC. Australian PHC policy is a mature system of UHC, and rates highly on the UHC service coverage index, compared to other OECD countries.^10^ However, a 2017 study of the health systems of eleven high-income countries ranked Australia second in overall performance but seventh on equity of access.^11^

 In this paper we draw on a five year study of PHC policy implementation in Australia. We examine Australian PHC policy, it strengths and weaknesses in addressing NCDs, and how it contributes to equity in access and health outcomes. We discuss key ideas, actor interests and structures shaping PHC policy, including two recent reforms intended to improve care for NCDs. The paper examines four key issues raised in international debates on UHC, in an Australian context:

- The roles of public or private funding or service delivery structures in UHC^8^,
- PHC responses to the growing impacts of NCDs^1^,
- Utilisation of comprehensive, selective or medical models of primary care^2^,
- And the extent to which UHC systems in practice will support universal and equitable access to PHC and equity in health outcomes.^2^

Our research question is: how do ideas, actor interests and structures in Australian PHC policy implementation affect the system’s ability to address NCDs, deliver equity of access to services according the need, and support equity in health outcomes?

 In the discussion we consider lessons the Australian experience may hold for international debates on UHC and jurisdictions seeking to implement UHC.

 We define PHC as comprehensive first-level care that incorporates but extends beyond primary medical care to include health promotion, disease prevention, community engagement and action to address SDH^12^; and public regulation of key social determinants of NCDs, such as the products and practices of tobacco, food and alcohol industries. However, we recognise that the term ‘PHC’ is used with different meanings. Thus for clarity, we will refer to services or models of care consistent with the above definition as ‘Comprehensive Primary Healthcare’ (CPHC); and regulatory measures as ‘public health regulation.’ We will use ‘PHC’ or ‘PHC policy’ as generic terms for the Australian system of first-level services including general practice (GP), community health and allied health services. We will use ‘primary medical care’ to describe more clinical approaches, which are common in Australia.

 We regard health inequalities determined by avoidable differences in socioeconomic conditions as socially unjust or unfair, and use the term ‘health inequities’ to signal this stance.^13^ We see that equity in access to PHC services occurs when people are able easily to use PHC services appropriate to their needs, regardless of their private ability to pay.^14^ Inequities in access are shaped by differences in the availability, affordability or acceptability of services.^15^ Health equity we define as the absence of systematic inequalities in health outcomes between groups with differing levels of social advantage or disadvantage.^16^

###  Australia’s PHC System – Setting for the Research 

 Australia’s federal system includes the national (or ‘Federal’) government and eight, regional State or Territory (hereafter, ‘State’) governments. In PHC policy, the Federal government subsidises use of GP services and some other forms of primary medical care through Medicare, a universal public health insurance scheme. The subsidy may cover the full cost to patients but GP services may charge additional fees. These and other out-of-pocket costs to citizens constitute around 18% of total health funding.^17^ Beyond Medicare, Federal and State governments (together or separately) fund various other PHC services. The majority of overall health funding goes to tertiary services meaning PHC is relatively under-funded, especially preventive and promotive forms of PHC.^18^

 Alongside Medicare, Federal policy also supports and subsidises a system of private health insurance (PHI), currently purchased by around half the population,^19^ which provides greater access to hospital care and various PHC services (other than Medicare and State-funded services) such as dental care, physiotherapy and other allied health services. Legislation prevents PHI coverage for GP services funded by Medicare.^19^

 GP services and other forms of primary medical care are delivered largely by health professionals operating as private businesses. Medicare does not regulate providers’ choice of location, leading to concentration of services in inner urban areas and deficits in regional, rural and remote locations despite their higher levels of need.^20^

 Non-government Aboriginal community-controlled health organisations (ACCHOs) and other community health services combine Medicare funding with targeted program funding from Federal and/or State agencies to provide a CPHC approach. The Community Health sector has declined over recent decades.^21^ The Federal government also funds primary health networks (PHNs) as regional PHC organisations operating within States, responsible for population health planning and commissioning to fill service gaps.

 The recent policy record on health promotion and public health regulation is mixed. A national health promotion policy was implemented between 2008 and 2013 but defunded after a change of government. In 2020, a new National Preventive Health Strategy is under development. National public health regulation of tobacco control is world leading^22^ and has contributed to significant decreases in smoking rates over the last 30 years.^23^ However, regulation of food and alcohol products is far more limited. As with other high-income countries, NCDs constitute the major burden of disease in Australia, and are more common amongst groups subject to disadvantage.^24^ Rates of obesity, a key risk factor for NCDs, continue to increase among Australian adults and, again, are inequitably distributed.^25^

 As in other countries,^26^ Australian policy adopts a predominantly biomedical view of health.^27^ Individualised views of health behaviours and risk factors for NCDs predominate, limiting promotion strategies to those focused on individual ‘lifestyle’ change and marginalising strategies that address social determinants by creating healthy social conditions.^27^ Australian PHC policy has also been shaped over time by contestation between social democratic values of universality, equity and public provision, and neoliberal values favouring market-based approaches and consumer choice.^28^ Social democratic values informed the creation of Medicare in 1984 under a centre-left Labor Party government.^29^ Neoliberal values informed expansion of PHI under a centre-right Liberal-National Coalition government in 2007.^19^

 The involvement of different levels of government, multiple public agencies, multiple funding mechanisms, private sector and community sector providers and advocacy groups makes for a complex PHC policy environment.

## Methods

###  Methodology

 We used a qualitative case study methodology suited to examining complex social phenomena in real-world settings.^30^ The case defined for the research was the structures and processes of Australian national and State government PHC policy implementation between 2008 and 2018. Data were collected between 2015 and 2018. Our aim was to understand how PHC policy implementation is likely to affect equity of access to PHC services and interact with SDH in shaping health equity outcomes.^31^

###  Use of Policy Theory

 Policy implementation enacts government decisions via mechanisms such as government departments, funding and regulatory tools and service provision.^32^ We adopted a critical view of policy implementation, not only examining what was done but also drawing on literature to consider what *else* could feasibly have been done and how such alternatives might have affected outcomes.

 To inform the empirical research we drew on Howlett et al^33^ who argue that public policy is determined through interactions between three main elements: defining *ideas*, policy *actors* and their interests, and organisational *structures*. We used these elements as a guiding framework for data gathering and primary analysis. We then used Cairney’s multi-theoretic approach^34^ to draw in further theoretical perspectives, testing these against our data, and delineating factors determining policy implementation in ways relevant to equity:

- *Ideas: *Recent institutionalist literature theorises how political beliefs and values held by influential policy actors enter into and shape policy implementation.^35^ These perspectives were useful in explaining the broad orientations and structural features of PHC policy.
- *Structures:* Hill and Hupe^32^ describe how policy implementation often occurs through structural and procedural relationships between funding agencies such as government departments and organisations delivering funded services. We drew on these perspectives to examine how methods of PHC policy regulation and funding affect service delivery and equity of access. Theory on multi-level governance^36^ was useful to consider the role of PHNs as a ‘meso-level’ of PHC governance.
- *Actors and interests:* Pluralist perspectives emphasise the role influential groups or organisations play in determining public policy by asserting their perceived interests.^33^

Analysis using these perspectives is woven through the results section below, and implications for future PHC policy favourable to equity are considered in the discussion.

###  Data Gathering and Analysis

*Mapping:* We searched online government sites and other sources to map the organisational, regulatory and policy structures in PHC at both national and State levels; including public, non-government and private sector organisations. We did our initial mapping in 2015 and updated this as required in the period up to 2019.

####  Monitoring the Policy Environment

 We conducted a narrative review of literature to identify key, recent changes in Australian PHC policy and examine likely effects on equity of access.^37^ We monitored the PHC policy environment by collating and reviewing relevant grey literature including: Ministerial statements, departmental websites, public or non-government organisations’ (NGOs) policy reports, and expert discussion on selected independent media sites. We tabulated date, source, and relevance of items discussing key aspects of PHC policy implementation relevant to our aim. These data were used to track events and gain critical perspectives on PHC policy from experts outside government.

####  Key Informant Interviews

 We identified actors in government agencies and NGOs closely involved in PHC policy implementation as prospective interviewees. We sought interviews with senior policy actors working in the national health department and with at least two policy actors in each State or Territory, from either the health department or a PHN. We conducted interviews with government policy actors from all but two State jurisdictions where invitations were declined, where we then interviewed a PHN representative.

 We also sought interviews with senior staff working for professional bodies and NGOs representing key target sectors including: GPs, nurses, other health professionals, rural health services, community health services including ACCHOs, healthcare consumers, and PHC research organisations. We interviewed a senior staff member from NGOs representing all the target sectors. A total of 30 semi-structured, 45-60 minute interviews were held, following methods approved by the Flinders University Research Ethics Committee. We approached prospective interviewees by email with an Information Sheet on the research. All interviewees provided written consent and were given an opportunity to review a transcript of their interview.

 The interview schedule for informants working nationally sought views on: the role of PHC in addressing NCDs; current issues for equity of access; funding and regulation; role of PHNs; involvement of PHI; roles of GP services, ACCHOs and community health services; current policy on health promotion; and recognition of or action on SDH in PHC policy. The schedule for State-based informants included additional questions about the national-State policy relationship; State-funded PHC services; and jurisdictional responses to inequities in access.

 Interview data were analysed thematically using NVivo software and an *a priori* coding framework developed by the authors based on our ‘ideas-actors-structures’ theory frame, knowledge of PHC policy, and our foci on implementation and equity. Further codes were added based on un-anticipated themes emerging from the data. Transcripts were imported into NVivo and thematically analysed using a coding framework that included codes for: key ideas shaping policy; key actors and organisations; implementation structures and processes; equity impacts or issues; references to SDH; role of public and private sector including PHI; national-State relations; health promotion; PHNs and workforce issues. Initial trial thematic coding of data was conducted independently by two researchers using NVivo and results compared to ensure reliability. Prior to commencing interviews we conducted a preliminary study on the involvement of the PHI industry in PHC in Australia.^19^ The major coding categories and associated themes resulting from data analysis are shown in Table 1.

**Table 1:** Major Coding Categories and Resulting Themes

**Major Coding Categories**	**Themes Resulting From Data Analysis**
Key ideas shaping policy	Dominance of biomedical and behavioural views of health NCDs as the major issue for PHC policy; GP-centric PHC system failing to properly manage NCDs resulting in avoidable hospitalisations Key risk factors for NCDs are purely matters of individual responsibility
Key actors and organisations	Health professional and private sector organisations strongly influence PHC policy, eg: Australian Medical Association, PHI providers, food and alcohol corporations Government health agencies control implementation Central position of private GPs in the PHC system ACCHOs and community health services a small element of the system
Implementation structures and processes	Most public healthcare funding goes to acute care, PHC under-funded Major funding structures shape policy implementation: Medicare, PHI, array of targeted funding Government agencies’ funding and regulatory practices determine ‘on-the-ground’ implementation; favour GPs delivering episodic primary medical care; prevent flexible governance at a local/regional scale ACCHOs and community health services draw on Medicare and targeted funding to deliver CPHC Implementation of GP-led chronic disease management and PHNs to improve NCD management Defunding of national health promotion agency and programs
References to SDH	Policy actors in PHC sector recognise impacts of SDH on health inequities and on demand for NCD care in the PHC sector ACCHOs try to address SDH as part of CPHC model
Health equity impacts or issues	Relative underfunding of PHC limits capacity for promotion and prevention Medicare favourable to equity of access by socioeconomic status, but fails to control inequitable distribution of services by location PHI contributes to inequities in access and health outcomes GP’s episodic primary medical care a poor model of care for NCDs ACCHOs provide access to culturally safe CPHC for Indigenous Australians; but are underfunded relative to need Devolved control over implementation to localised governance structures has potential to meet local needs more effectively Lack of public health regulation of food and alcohol industries contributes to NCDs, health inequities, demand on PHC sector
Role of public and private sector including PHI	PHC policy implementation delivered via a highly complex mix of public and private sector structures Mix of public and private structures reflects influence of social-democratic and neoliberal governments over time
National-State relations	Divisions of responsibility for healthcare policy between Federal and State governments results in implementation problems, eg, poor interface between hospitals and PHC providers
Health promotion	Lack of policy support and funding for health promotion policies at the time the research was conducted Unwillingness to regulate food and alcohol industries
PHNs and workforce issues	PHNs lack funding and autonomy to carry out their role effectively Complex array of targeted funding undermines workforce security and create high administrative demands on ACCHOs

Abbreviations: NCD, non-communicable disease; PHC, primary healthcare; GP, general practice; PHI, private health insurance; CPHC, comprehensive primary healthcare; ACCHOs, aboriginal community-controlled health organisations; PHNs, primary health networks; SDH, social determinants of health.

####  Integration of Data From Different Sources

 After coding was completed, discussion in team meetings was used to delineate key findings. We drew on summary reports of themes and indicative quotes resulting from data coding and applied our theory-informed ‘ideas, actors and structures’ framework over a series of meetings to integrate analyses of data from our several sources. In this way we arrived at agreed views on key ways in which ideas, actors and interests, and organisational structures shaped PHC policy implementation during the period examined, in ways likely to affect care for NCDs, equity of access according to need and equity in health outcomes.

## Results

 Our analysis showed several main elements of Australian PHC policy shape its strengths and weaknesses to achieve UHC, address NCDs, deliver equity of access, and contribute to health equity. These include particular dominant ideas shaping the policy environment (ideas); individuals and organisation exercising policy influence (actors); three structural approaches to funding and regulation, and two structural reforms intended to improve care for NCDs (structures). These elements are reflected in the headings below.

###  Dominant Ideas in Recent PHC Policy 

 Biomedical and behavioural views of health are dominant in Australian health policy documents.^27,28^ Our research shows that PHC policy implementation also occurs largely via delivery of a biomedical and behavioural model of care. Contestation between social democratic and neoliberal values was also present, especially in the combination of public or private insurance structures in the Australian system.^19^ In addition, one set of ideas emerged as strongly influencing policy-makers views about problems to address in Australian PHC policy: that the GP-centric PHC system was failing to properly manage NCDs among the Australian populace and to prevent costly, avoidable hospitalisations.^38^ Thus, policy reforms in this period were focused on improving GP services’ management of patents with NCDs in order to reduce avoidable hospitalisations. These ideas are shown in the report of a Health Minister-appointed PHC policy advisory group in 2015:

*“Australia is experiencing increasing rates of chronic and complex conditions, which challenge our current primary healthcare system … Patients with chronic and complex conditions are high users of health services … Currently, primary healthcare services in Australia for this patient cohort can be fragmented, and often poorly linked with secondary care services*.”^38^

 Concurrently, individualised, behaviourist views of risk factors for NCDs such as diet, exercise and alcohol consumption (ie, that these are purely matters of individual responsibility) held by conservative national governments in power since 2013, led to a withdrawal of investment in previously established community health promotion policies and unwillingness to regulate to reduce adverse health equity impacts of the food and alcohol industries.

 Many of the health department policy actors we interviewed recognised the impacts of SDH on (inequities in) NCDs. However, this understanding was largely subjugated to the more individualised and biomedical conceptions of health held by elected decision-makers and other powerful lobby groups, or embedded in current institutional practices, in determining policy directions.^27^

###  Key Actors Exercising Policy Influence

 Our findings indicate that, outside government, the key organisational actors exercising influence on PHC policy in the period examined were medical professionals’ representative organisations, PHI companies, and food or alcohol industry corporations.

*“…change is difficult because of the power of vested interests … private health insurance, the AMA [Australian Medical Association], Medicines Australia and the Pharmacy Guild. I think those four between them effectively control what happens in healthcare in Australia”* [I17-technical expert].

*“…we have been world leaders in addressing smoking … we’ve got into trickier territory with obesity … there are massive interests in the food industry, in the alcohol industry resistant to any change” *[I5-NGO].

 According to our research, areas of PHC policy implementation where these organisations have exercised influence include: the dominant positioning of private GP practices in policy delivery – as one informant put it, ‘*both this government and previous Labor governments [put] GPs in the central position as gatekeeper*’; lack of effective public health regulation of the food and alcohol industry to reduce NCD risk; and an increased role of PHI in PHC policy (although not including coverage of Medicare funded GP services).^19^

 Several informants argued that Medicare could use its control of funding to regulate availability of GP services and other forms of primary care in underserved regional, rural or remote locations; but does not do so because the AMA would oppose it.

*“If [Medicare] tried to do that then, you know, that is the one thing that the AMA would actually encourage a doctors’ strike over and … it would never happen because of that” *[I7-NGO].

 The lack of regulatory power within Medicare to control the distribution of PHC services leads to a concentration of services in relatively healthy inner urban areas and undersupply of services in outer suburban, regional, rural and remote areas.^20^ Our research also highlighted the power exerted by Australian government health agencies over the processes of PHC policy implementation, through their control over funding and regulation of services. As reported below, these approaches have implications for the type of PHC service delivered and capacity of services to meet community needs.

###  Funding Structures: Medicare and Private Health Insurance 

 Medicare is a UHC system and the major institutional feature of Australian health policy; using public resources to fund universal, free or subsidised PHC services. This structure is broadly favourable to equity of access to GP services,^39,40^ the most highly utilised form of PHC, and to subsidised medicines, although some population groups still face inequities in access.^20^ Most informants expressed strong support for Medicare in these terms, for example:

*“ … compared to many other countries Australia does very well [on equity of access]. We have a system of universal health coverage through Medicare [offering] subsidised, and in many cases free, access to General Practice services [and…] to other specialist services”* [I10-NGO].

 However, four structural features of Medicare funding for PHC were prominent in the data as contributing to sub-optimal care for NCDs in the PHC system, contributing to additional costs in acute care services.

1. The relatively low level of public funding for PHC compared to acute care services.
2. Medicare positioning privately-operated GP services offering primary medical care as the main vehicle for service delivery.
3. Medicare being structured to fund PHC services on a fee-for-service basis, with payments linked to many separate items of episodic care.
4. The structural separation between nationally controlled Medicare funding for PHC services, and the State-managed hospital system, resulting in poor information sharing and patient transitions between the two systems.

The result of 2 and 3 together was seen to be that Medicare funding for PHC goes predominantly to GPs offering episodic primary medical care, while optimal care for chronic NCDs requires continuity of care and multi-disciplinary care teams.^41^ Key policy actors identified this perceived mismatch as the underlying cause of sub-optimal care for NCDs in the PHC system, and thus of avoidable hospitalisations.^38^ Resultant policy reforms (discussed below) focused only on addressing point 3 and 4 within the confines of the existing system of GP services and its relationship with State-managed tertiary services; and avoided the systemic funding issues and dominance of GPs as per points 1 and 2.

 However, many of our informants presented a broader view of a desirable PHC policy response to NCDs; acknowledging the weaknesses in fee-for-service funding to GPs, but also highlighting the overall under-funding of PHC as a problem (point 1). As one State health department actor said:

*“Normally acute services get the bigger bucket of money … it’s a vicious circle, you’ve got people fronting up at acute services that need admission, but I don’t have enough resources [in PHC] to try and stop them getting acute*”[I25-state government].

 Several informants also argued that the GP-centric nature of Medicare (point 2), and of PHC policy reforms to tackle NCDs, marginalises the potential role of CPHC services and nurses in providing patient-centred, multi-disciplinary forms of care well-suited to needs of patients with NCDs. We will examine data on the role of CPHC services in section 3.5.

 Furthermore, our analysis identified several further concerns about Medicare and other structural features of Australian PHC policy not included in the four points above and not considered in policy reforms. All are relevant to current international debate on UHC, including questions of prevention of NCDs, equity, selective coverage and the role of PHI.

 First, analysis of our data suggested that the dominant focus on GP services within reforms to improve responses to NCDs, plus neoliberal beliefs about health behaviours (ie, that they are primarily matters of individual responsibility) combined to severely limit primary disease prevention and health promotion policies during the period we examined. While some NCD screening programs were supported other strategies, such as public health regulation of food and alcohol sectors, were not. Such measures could significantly reduce risk factors such as obesity and incidence of NCDs, which contribute to demand in the PHC sector.

*“I think that at the national level, our federal Department of Health has really dropped the ball on preventive health and health promotion. I think the [subsequently defunded] National Preventive Health Agency was a terrific initiative … and why do we need a national preventive health agency? To keep people well. To prevent chronic disease” *[I10-NGO].

 Second, as noted earlier, Medicare’s universal coverage excludes several forms of PHC service such as dental care, physiotherapy and allied health services. Australia’s PHI providers offer coverage for all these services, to those who can afford it, in a way that requires co-payments. Federal or State governments also use targeted funding to extend access to some such services. This arrangement means the PHC system overall struggles to provide preventive care for NCDs such as dental caries and lower back pain,^42,43^ and restricts access for those who can’t afford PHI to relevant services such as primary dental care and physiotherapy. In dental care, for example, the role of PHI contributes to significant inequities in timely access to care and health outcomes, resulting in thousands of avoidable hospitalisations.^44,45^ As one informant described:

*“[Dental care] is the other major area where there’s massive inequalities… if you want an example of a two tiered system we’ve got it in dental” *[I5-NGO].

 Several of our informants also highlighted the structural features of Medicare PHC funding that lead to a concentration of GP services in urban areas, and lesser availability in regional, rural and remote locations.^20^

*“…the main problem in rural health is that you can only access Medicare as a citizen if you can access a health professional. In rural and … remote areas where there is an undersupply of the workforce that necessarily leads to underservicing and an underutilisation of Medicare” *[I7-NGO].

 This contributes to significant inequities in Medicare PHC spending per person between major urban centres and country areas,^46^ and limits the capacity of the PHC system to prevent or manage the higher rate of NCDs in areas outside of Australia’s major cities.

 In summary, although Medicare as a system of UHC does support equity of access to primary medical care by reducing financial barriers, our research shows this is not enough to tackle the growing challenge of NCDs in an optimal and equitable manner.

###  Two PHC Structural Policy Responses to Chronic NCDs

 The PHC reforms undertaken in our study period focused on Medicare’s fee-for-service structure, and improving primary medical care in order to manage chronic NCDs and reduce avoidable hospitalisations:

*“The general direction in terms of primary care is to try and develop greater capacity within this sector to actually manage greater acuity, people with greater acuity who might otherwise need to go to hospital and of course hospital avoidance, emergency department diversion, but in order to do those things you have to build capability within the primary healthcare sector”* [I18-state government].

 Two main policy reforms emerged: funding incentives for GP services to better ‘manage’ patients with chronic NCDs; and reform of existing regional PHC organisations – PHNs.

###  General Practice-Led Chronic Disease Management 

 During the period researched the Federal government introduced two reforms to incentivise PHC services to shift from episodic care to a model of continuous, multi-disciplinary care for patients with NCDs^37^: Medicare funding for ‘chronic disease management’ and trial of a new capitated funding structure – ‘Healthcare Homes’ – outside Medicare.^47^ Some informants saw these changes as favourable to equity of access for people with these conditions,^41^ providing an alternative to episodic primary medical care:

*“Because the needs of people … we’re talking about where there are real inequities in access or outcomes are not the ones that need just an episodic-type management”* [I29-State Government].

 The Healthcare Homes trial was taken up by some GP services, larger multidisciplinary PHC practices and ACCHOs; including services operating in areas of disadvantage. Little evidence is yet available on outcomes^48^ but given the model of care it aims to provide – one that moves toward a CPHC model – again, it was seen as a potential gain in system capacity to provide effective care for people with NCDs.

 However, we identified several concerns about Healthcare Homes and chronic disease management. In particular, informants noted that both programs are simply ‘add-ons’ to the continuing Medicare system of fee-for-service funding for episodic primary medical care, which continues to be GPs’ main source of income and model of practice. Thus uptake by GPs may be limited:

*“We do have a few very engaged private general practices … participating in the Healthcare Homes … they’re actually focusing on outcomes for their patients … But of course a lot of GPs don’t do that at all. They do just literally have people coming through the door” *[I24-State government].

 Another concern was that GPs may struggle to coordinate multi-disciplinary care in country areas where allied health services are less available, or patients’ access depends on PHI.

 Given these issues, some respondents argued that resources expended on the two programs under discussion would be better spent on strengthening and extending CPHC services, rather than trying to redirect the entrenched practices of many GPs; for example:

*“Many of the elements of the Healthcare Homes is exactly how Aboriginal primary healthcare services in the NT already deliver their chronic disease care. We have enrolled patients, we have electronic health records, we have care plans, we have structured recalls, [and] we monitor the data very closely” *[I24-state government].

###  Primary Health Networks

 PHNs are the most recent of several iterations of regional primary care organisation in Australia, others being Divisions of GP and Medicare Locals, and were introduced in 2014-2015 following election of a conservative Coalition Federal government. Their general role is to plan, coordinate and (if needed) commission PHC services at a regional level; a role endorsed by our informants as useful and favourable to equity, for example:

*“[PHNs have…] a key regional role around planning and co-ordination of health services alongside state-funded services … and commissioning and working with local stakeholders to actually not only ensure a continuity of service delivery but a more targeted or better targeted and co-ordination of services on the ground … so that it’s better meeting the needs of the population in that region” *[I1-Government].

 However, during the period researched, PHN guidelines prioritised the same narrow goals motivating other reform: better management of NCDs and reduced avoidable hospitalisations, with a focus on improving patient transitions between (Federally funded) primary medical care and (State managed) public hospitals.^49^ Our findings indicated that such improvements would likely benefit patients, but that this narrow policy focus also severely restricts PHNs broader, potential role in population health planning, workforce development, health promotion, supporting CPHC services, or brokering inter-sectoral action on SDH.^28^ As one informant expressed it:

*“I think the problem is that the concept [of PHNs] is fine but they keep being given very specific, directed funding which then doesn’t necessarily give them sufficient leeway to actually address inequities” *[I24-state government].

 In summary, two policy reform implemented in Australia intended to address the weaknesses of fee-for-service, episodic primary medical care as a model of care for NCDs appear to be severely limited by a focus on reforming biomedically-oriented GP services and hospitals, with a concomitant lack of attention on other systemic issues affecting NCDs, as covered in the section above on funding structures.

###  Targeted Funding Structures and the Role of CPHC Services

 Outside of Medicare and PHI, Federal and State governments (jointly or separately) provide targeted funding for a wide range of PHC services or programs, such as:

- Funding GPs to offer after-hours care or work in rural areas,
- Disease or risk-factor focused interventions, and
- Specialised services: eg, child and maternal health, sexual health, family health, mental health, alcohol and other drugs, Aboriginal and Torres Strait Islander health and health promotion.

In addition, multi-disciplinary CPHC services such as community health services and ACCHOs draw on Medicare to provide primary medical care, but must combine this with targeted funding sources to achieve their comprehensive approach. As one informant noted:

*“There’s not a lot of room in the current [Medicare] funding structures for… models that might address better community need and social determinants of health” *[I4-NGO].

 These CPHC services often will incorporate specialised services as noted above within their operations. Several informants described a CPHC model as very well-suited to prevention of, and effective care for NCDs, taking account of social factors; for example:

*“Yeah, well, that [CPHC approach is] what would happen ideally, across the sector. We know it’s difficult and there are barriers to doing that but, yes, that holistic approach, the prevention, the awareness, as well as treatment and management of conditions, they all work towards better outcomes” *[I8-NGO].

 Thus, targeted funding in Australian PHC policy adds to Medicare’s universal coverage by funding the delivery of both CPHC services and specialised PHC services targeting particular groups; groups whose need may not be well catered for by ‘generic’ GP services. However, compared with Medicare-funded GP services, these services comprise only a tiny part of the PHC sector. Furthermore, triangulation between our three forms of data also indicates that current targeted funding practices create systemic problems. A fragmented array of relatively small, competitive and prescriptive funding programs across two levels of government has created a very difficult environment for many funded services. Challenges include financial and workforce insecurity, high administrative burden, service duplication, prescriptive regulation, and one-size-fits-all programs that do not suit local conditions or needs. Many ACCHOs delivering services in Indigenous communities are adversely affected by these conditions.^50^ All of these issues are likely to detract from the capacity of CPHC and specialised PHC services to contribute to effective, equitable prevention of, and care for NCDs.

###  Potential for Structural Reform

 The inclusion of theory on implementation^32^ and multi-level governance^36^ in our analysis framework led us to examine the regulatory relationship between public funders and service providers, and the level of governance at which the use of PHC funding is determined. Most informants from PHNs, state-funded PHC services, community health services and ACCHOs argued that increased *local control* over PHC policy resources would support more flexible and efficient use of resources to improve services. They largely saw the current fragmented array of targeted funding as an available opportunity for such reform, but their critique is also relevant to the structure of Medicare. For PHNs, benefits of increased local flexibility and control over resources were conceived in terms of improved allocation of resources to match services and workforce to local needs:

*“Commonwealth [is] giving us funds typically still on a program allocation basis … which again is counterintuitive to a whole-of-population commissioning approach [where…] you would leave us to determine what the community needs were, what the service and system needs were and then look at how we use our funds globally to address those needs”* [I21-PHN].

 For community health services and ACCHOs benefits were conceived in terms of enhanced capacity to deliver a CPHC model of service, adapted to local community needs.

*“…the joy about Community Health is it ought to be flexible enough to be able to adapt to the local community and the changing needs of the local community” *[I28-State government].

 One approach suggested by several informants to support the desired flexibility – whether for a PHN or a service provider – was to shift away from activity-based funding that allocates money on a cost-per-service basis, to a block funding approach, which allocates funding for specified purposes but allows the funded agency more local flexibility in how those purposes are achieved.^51^ The use of an existing (minor) block funding scheme by some rural health services to fund local services and engage communities was noted as evidence of the merits of this approach:

*“Our Deputy Secretary visited a service in [country town] … they’ve become a multipurpose service and they can use their funding in a very flexible way … They’ve got a mental health worker on board … They’ve got a youth engagement worker … a team of about 350 volunteers. People running diabetes support groups … they’ve had some incredible outcomes in terms of a tangible reduction in alcohol and drug use. An increase [in] school retention and no suicide of a young person in 20 years”* [I28-State government].

 Thus, our findings indicate that forms of PHC funding providing greater local control and flexibility have the potential to address some of the weaknesses or limitations of existing Medicare funding for GP services and of current approaches to targeted funding. However, to ensure equity, measures would still be required to match funding to assessed needs in different regions.

## Discussion

 The Australian experience demonstrates that UHC is likely to improve equity of access to PHC services compared to a situation where access depends on private ability to pay. With Medicare, Australian health policy meets objectives identified in the current vision for UHC^3,4^ for a system of coverage delivering universal, affordable access to some form of PHC – in this case, primary medical care; across socioeconomic status levels. Australia positions GP services and selected other forms of primary medical care at the centre of PHC policy. Medicare acts, in effect, as a monopoly, fee-for-service purchaser of these services, controlling costs to consumers to some extent. This system is supplemented with targeted public funding for services to meet needs of particular population groups and functions not covered by fee-for-service GPs.

 However, notwithstanding these achievements, our research shows that ideas, actors and structures interact to shape Australian PHC policy in ways that limit its capacity to address the rising challenge of NCDs, deliver equitable access according to need, and support equitable health outcomes. These lessons are relevant to international debate and jurisdictions implementing UHC. For example, our analysis indicates that pervasive, biomedical ideas of health, the policy influence of medical professionals and the structural features of Medicare all play a part in sustaining a PHC system based on episodic primary medical care, with services distributed in ways that disadvantage outer-suburban parts of, and areas outside, Australia’s major cities. The limitations of episodic primary medical care as a model of care for NCDs was a core idea driving policy reforms in the period examined. However, the structural centrality of GPs within Medicare and their policy influence appear to have limited reforms to strategies that, again, place GPs at the centre and overlook other salient reform opportunities.

 Further, neoliberal political values and the policy influence of the PHI industry have shaped the current structure of public and PHI system in Australia; contributing to significant inequities in access to services such as dental care, and inequities in NCDs such as dental caries. This experience is consistent with broader warnings about combining public and private systems in general health policy; notably development of inequitable, two-tier systems of care.^52^

 The dominance of primary medical care and marginal role of CPHC services, absence of policy support for health promotion and lack of public health regulation of food and alcohol industries all acted to limit action on SDH (and of NCDs in particular) in PHC policy during the period examined.

 Our research indicates that a number of ideational, actor-centred and structural features are likely to be useful in other jurisdictions to optimise a system of UHC for PHC that: addresses the challenge of NCDs, delivers equity of access to care according to need, and supports equity in health outcomes across geographically and ethnically diverse populations. These are shown in Table 2.

**Table 2:** Recommended Features of Universal Health Coverage for Primary Healthcare

**Ideas**	PHC policy-making based on a broad biopsychosocial model of health Commitments to principles of universal access according to need, health equity and ‘prevention is better than cure’ System based on an understanding of the strong influence of environmental, social and commercial determinants on health
**Actors**	Policy decision-making processes that limit the influence of sectional groups with financial interests in policy settings, including medical professionals, PHI, and the tobacco, food and alcohol industry sectors
**Structures**	A public UHC scheme, adequately funded through progressive taxation measures, committed to equity of access to all essential PHC services including dental Regulatory measures and incentives to ensure a distribution of services and personnel that matches community needs in different areas Universal, affordable access to block-funded, multi-disciplinary CPHC services that: flexibly tailor services to local conditions and needs; provide coordinated care for chronic conditions; promote health; engage local communities; advocate for their health and offer culturally safe services to Indigenous peoples and other minority groups Support for general practitioners as an essential part of a multi-disciplinary PHC workforce including nurses, allied health professionals, and community health workers; with secure employment conditions Supplementary targeted funding to ‘top-up’ service responses in particular locales (according to need) or rapidly scale up responses to emerging health issues Public health regulation to limit impacts of corporatised food, alcohol and tobacco sectors on NCDs Regional PHC organisations or regional health authorities with a mandate and resources to undertake population health planning and workforce planning, ensure coordination between primary, secondary and tertiary care services, and broker inter-sectoral partnerships to address SDH

Abbreviations: NCDs, non-communicable diseases; PHC, primary healthcare; UHC, Universal health coverage; PHI, private health insurance; CPHC, comprehensive primary healthcare; SDH, social determinants of health.

###  Limitations

 It was beyond the scope of the research to engage directly with PHC service providers or service users and thus to incorporate their perspectives in this paper. We do not address policy influences on determinants of health such as income, education, employment or housing as these fall largely outside the purview of health system PHC policy. We do recognise that a comprehensive PHC approach would incorporate consideration of these sectors. There are many lessons already from the community health movement^53,54^ about ways in which PHC policy implementation can be coordinated effectively with the work of other sectors to address these broader SDH. These need to be further understood and in particular the ways in which the Australian PHC sector can transition to more effective comprehensive models of care.

## Conclusion

 Policy choices about structures and processes to implement UHC are important to equitable outcomes. Institutionalised structures for funding and delivery and powerful sectoral interests – once in place – can act as barriers to systemic policy change. Although Australian PHC policy has major features favourable to equity of access, other features detract from the capacities of the system to take preventive action on, and better manage NCDs, deliver equity of access according to need, and support equity in health outcomes. Countries aiming to achieve these goals can learn from strengths and weaknesses in the Australian system.

## Acknowledgements

 We would like to acknowledge and thank our colleagues on the Centre of Research Excellence on the Social Determinants of Health Equity research team, members of the project’s Critical Policy Reference Group, and all those who participated in the research.

## Ethical issues

 The research reported was approved by the Flinders University Social and Behavioural Research Ethics Committee.

## Competing interests

 MF reports grants from National Health and Medical Research Council, during the conduct of the study.

## Authors’ contributions

 MF contributed to research design, led data gathering and analysis, and led writing of the paper. TF contributed to research design, data gathering and analysis, and reviewed draft versions of the paper. TM contributed to research design and data analysis, and reviewed draft versions of the paper. SF co-led the research team, led research design, contributed to data analysis, and reviewed draft versions of the paper. FB co-led the research team, led research design, contributed to data analysis, and reviewed draft versions of the paper.

## Funding

 The research reported in this article was conducted through the Centre of Research Excellence on the Social Determinants of Health Equity, funded by the Australian National Health and Medical Research Council (GNT1078046). The funder had no role in: study design; collection, analysis and interpretation of data; writing of the article; or the decision to submit it for publication.

## Authors’ affiliations

^1^Southgate Institute for Health, Society and Equity, Flinders University, Adelaide, SA, Australia. ^2^College of Medicine and Public Health, Flinders University, Adelaide, SA, Australia. ^3^The George Institute for Global Health, Sydney, NSW, Australia. ^4^RegNet School of Regulation and Global Governance, Australian National University, Canberra, ACT, Australia.
